# pySPACE—a signal processing and classification environment in Python

**DOI:** 10.3389/fninf.2013.00040

**Published:** 2013-12-24

**Authors:** Mario M. Krell, Sirko Straube, Anett Seeland, Hendrik Wöhrle, Johannes Teiwes, Jan H. Metzen, Elsa A. Kirchner, Frank Kirchner

**Affiliations:** ^1^Robotics Group, Faculty 3 - Mathematics and Computer Science, University of BremenBremen, Germany; ^2^Robotics Innovation Center, DFKI GmbHBremen, Germany

**Keywords:** Python, neuroscience, EEG, YAML, benchmarking, signal processing, machine learning, visualization

## Abstract

In neuroscience large amounts of data are recorded to provide insights into cerebral information processing and function. The successful extraction of the relevant signals becomes more and more challenging due to increasing complexities in acquisition techniques and questions addressed. Here, automated signal processing and machine learning tools can help to process the data, e.g., to separate signal and noise. With the presented software pySPACE (http://pyspace.github.io/pyspace), signal processing algorithms can be compared and applied automatically on time series data, either with the aim of finding a suitable preprocessing, or of training supervised algorithms to classify the data. pySPACE originally has been built to process multi-sensor windowed time series data, like event-related potentials from the electroencephalogram (EEG). The software provides automated data handling, distributed processing, modular build-up of signal processing chains and tools for visualization and performance evaluation. Included in the software are various algorithms like temporal and spatial filters, feature generation and selection, classification algorithms, and evaluation schemes. Further, interfaces to other signal processing tools are provided and, since pySPACE is a modular framework, it can be extended with new algorithms according to individual needs. In the presented work, the structural hierarchies are described. It is illustrated how users and developers can interface the software and execute offline and online modes. Configuration of pySPACE is realized with the YAML format, so that programming skills are not mandatory for usage. The concept of pySPACE is to have one comprehensive tool that can be used to perform complete signal processing and classification tasks. It further allows to define own algorithms, or to integrate and use already existing libraries.

## 1. Introduction

Time series are recorded in various fields of neuroscience to infer information about neural processing. Although the direct communication between most parts of the nervous system is based on spikes as unique and discrete events, graded potentials are seen as reflections of neural population activity in both, invasive and non-invasive techniques. Examples for such time series come from recordings of local field potentials (LFPs), electroencephalography (EEG) or even functional magnetic resonance imaging (fMRI).

Common characteristics of time series data reflecting neural activity are: (i) a high noise level (caused by external signal sources, muscle activity, and overlapping uncorrelated brain activity) and (ii) a large amount of data that is often recorded with many sensors (electrodes) and with a high sampling rate. To reduce noise and size the data are preprocessed, e.g., by filtering in the frequency domain or by averaging over trials and/or sensors. These approaches have been very successful in the past, but the solutions were often chosen manually, guided by the literature, visual inspection and in-house written scripts, so that possible drawbacks remain. It is still not straightforward to compare or reproduce analyses across laboratories and the investigator has to face many choices (e.g., filter type, desired frequency band, and respective parameters) that cannot be evaluated systematically without investing large amounts of time. Another (sometimes) critical issue is that the data might contain so far undiscovered or unexpected signal components that might be overseen by the choice of the applied data analysis. False or incomplete hypotheses can be a consequence.

While there is no single solution to all of these problems, recent tools for neuroinformatic purposes can help to compensate these drawbacks, especially when made available open source, by providing a common ground that everyone can use. As a side effect, there is the chance to enhance the reproducibility of the conducted research, since researchers can directly exchange how they processed their data based on the respective specification or script files. Based on the commercial software package Matlab, there are open source toolboxes existing, like EEGLAB (Delorme and Makeig, 2004) and FieldTrip (Oostenveld et al., 2011) for MEG, EEG, and SPM (http://www.fil.ion.ucl.ac.uk/spm/) especially for fMRI data. Respective Python libraries are for example PyMVPA (Hanke et al., 2009), OpenElectrophy (Garcia and Fourcaud-Trocmé, 2009), and the NIPY software projects (http://nipy.org/). Additional help comes from an increasing number and complexity of signal processing and classification algorithms that enable more sophisticated processing of the data. However, this is also considered as a problem, since it also demands (i) available tools where the signal processing algorithms can be directly compared (Sonnenburg et al., 2007; Domingos, 2012) and (ii) to close the still existing large gap between developer and user. On the other hand, the success of applications using automatically processed and classified neurophysiological data has been widely demonstrated, e.g., for usage of brain-computer interfaces (Lemm et al., 2004; Bashashati et al., 2007; Hoffmann et al., 2008; Kirchner et al., 2013; Seeland et al., 2013) and classification of epileptic spikes (Meier et al., 2008; Yadav et al., 2012). These applications demonstrate that automated signal processing and classification can indeed be used to directly extract relevant information from such time series recordings.

With the software pySPACE (*S*ignal *P*rocessing *A*nd *C*lassification *E*nvironment written in Python) we introduce a modular framework that can help (neuro)scientists to process and analyze time series data in an automated and parallel fashion. The software supports the complete process of data analysis, including processing, storage, and evaluation. No individual execution scripts are needed, instead users can control pySPACE via text files in the YAML format (Ben-Kiki et al., 2008), specifying what data operation should be executed. The software was particularly designed to process windowed (segmented) time series and feature vector data, typically with classifiers at the end of the processing chain. For such supervised algorithms the data can be separated into training and test data. pySPACE is, however, not limited to this application case: data can be preprocessed without classification, reorganized (e.g., shuffled, merged) or manipulated using own operations. The framework offers automatic parallelization of independent (not communicating) processes by means of different execution back-ends, from serial over multicore to distributed cluster systems. Finally, processing can be executed in an offline or in an online fashion. While the normal use case is concerned with recorded data saved to a hard disk (and therefore offline), the online mode, called *pySPACE live*, offers the application-directed possibility to process data directly when it is actually recorded without storing it to hard disk. We refer to this processing here as *online* due to the direct access in contrast to *offline* processing where the input data is loaded from a hard disk.

To tackle the problem of an increasing number of signal processing algorithms, additional effort was put into the goal of keeping pySPACE modular and easy-to-extend. Further algorithms can be added by the advanced user; the algorithms will be automatically included in the collection of available algorithms and into the documentation. Furthermore, the software is capable of using existing signal processing libraries, preferably implemented in Python or using existing wrappers to other languages like C++. So far, interfaces are implemented to external classifiers [from Scikit-learn (Pedregosa et al., 2011) and LibSVM (Chang and Lin, 2011)], the Modular Toolkit for Data Processing [MDP; (Zito et al., 2008)], WEKA (Hall et al., 2009), and MMLF (http://mmlf.sourceforge.net/). Core functionality of pySPACE uses the Python libraries NumPy (Dubois, 1999) and SciPy (Jones et al., 2001).

pySPACE was implemented as a comprehensive tool that covers all aspects a user needs to perform the intended operations. The software has a central configuration where the user can optionally specify global input and output parameters and make settings for individual paths to external packages as well as setting computational parameters. The processing is then defined in individual specification files (using YAML) and the framework can be executed with the respective operation on several datasets at once. This functionality is not only provided for internal algorithms, but can also be used with external frameworks like WEKA and MMLF. For the basic signal processing algorithms implemented in pySPACE, we adopted the node and flow concept of the MDP software (Zito et al., 2008)^1^ together with basic principles that were introduced together with it. Currently, more than 100 of such signal processing nodes are integrated into pySPACE. These nodes can be combined and result in numerous different processing flows. Different evaluation schemes (e.g., cross validation and metric calculation) are provided and different evaluation results can be combined to one output. This output can be explored using external software or by using a graphical user interface provided within pySPACE.

For basic preprocessing, analysis and visualization of electrophysiological data the existing neuroinformatics tools are probably sufficient. However, a drawback of most frameworks is that they focus on the preprocessing and a machine learning part is often missing (or vice versa). Furthermore, they do not enable a simple configuration and parallel execution of processing chains. To enable a connection to existing tools, pySPACE supports feature vector data in ARFF and CSV file format and can read and segment time series data in CSV, BrainProducts eeg, EEGLAB set, and EDF file format. As soon as several datasets have to be processed automatically with a set of different processing algorithms (including classification) and numerous different parameter choices, pySPACE is probably the better choice in comparison to the other tools. Additionally, the capability to operate on feature vector data makes pySPACE useful for a lot of other applications, where the feature generation has been done with other tools. To the best of our knowledge, pySPACE is unique in its way of processing data with special support of neurophysiological data and with its amount of available algorithms.

The structural concepts of pySPACE will be outlined in section 2. In section 3 we will shortly describe how the software is interfaced followed by the requirements for running it (section 4). Finally, we will give some application examples (section 5) and discuss related work (section 6).

## 2. Structure and principles

The software package structure of pySPACE was designed in order to be self-explanatory for the user. Core components in the main directory are run containing everything that can be executed, resources where external and internal data formats and types are defined, missions with existing processing algorithms the user can specify, and environments containing infrastructure components for execution. How to run the software is described in sections 3 and 5. The other packages and their connections are described in the following.

### 2.1. Data

**Figure 1:**
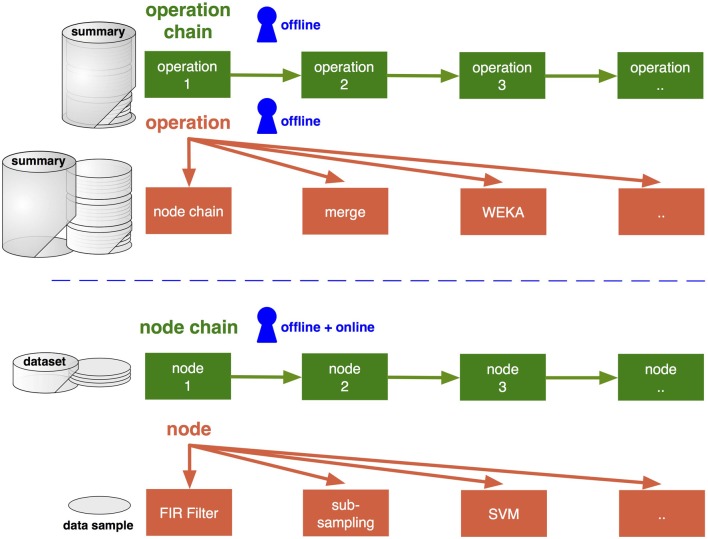
**High-level and low-level processing types (upper and lower part) and their connection to the data granularity (summary, dataset, sample).** Access levels for the user are depicted in blue and can be specified with YAML files (section 3.2). Only low-level processing can be performed online. For offline analysis, it is accessed by the node chain operation. For the operations and nodes several different algorithms can be chosen. Algorithms are depicted in orange (section 2.2) and respective infrastructure components concatenating these in green (section 2.3).

As a good starting point, one can look at the way the data are organized and handled within the software, including ways to load data into the framework and how the outcome is stored. Data are distinguished in pySPACE by its granularity: from single data samples to datasets and complete summaries (defined in the resources package), as explained in the following. They require at the same time different types of processing which are subsequently described in sections 2.2 and 2.3 and depicted in Figure 1.

Four types of *data samples* can occur in pySPACE: the raw data stream, the windowed time series, feature vectors and the prediction vector. A data sample comes with some metadata for additional description, e.g., specifying sensor names, sampling frequency, feature names or classifier information. When loading a *raw data stream* it is first of all segmented into *windowed time series* data. Windowed time series have the form of two-dimensional arrays with amplitudes sorted according to sensors on the one axis and time points on the other. *Feature vectors* are one-dimensional arrays of feature values. In a *prediction vector* the data sample is reduced to the classification outcome and the assigned label.

For analysis, data samples are combined to *datasets*. In pySPACE, a dataset is defined as a recording of one single experimental run, either as streamed data or already preprocessed as a set of the corresponding time series windows, or as a loose collection of feature vectors. It also has metadata specifying the type, the storage format, and information about the original data and preceding processing steps. For each type of dataset, various loading and saving procedures are defined. Currently supported data formats for loading streaming datasets are the comma separated values (.csv), the European Data Format (.edf), and the two formats specifically used for EEG data which are the one from Brain Products GmbH (Gilching, Germany) (.eeg) and the EEGLAB (Delorme and Makeig, 2004) format (.set). With the help of the EEGLAB format several other EEG data formats can be converted to be used in pySPACE. For cutting out the windows from the data stream, either certain markers can be used or stream snippets with equal distance are created automatically. For supervised learning, cutting rules can be specified to label these windows. Feature vector datasets can be loaded and stored in csv files or the ARFF (Attribute-Relation File Format) format, which is, e.g., useful for the interface to WEKA (Hall et al., 2009).

Groups of datasets, e.g., experimental repetitions with the same subject or different subjects, can be combined to be analyzed and compared jointly. Such dataset collections are called *summary* in pySPACE. Summaries are organized in folder structures. To enable simple evaluations, all single performance results in a summary are combined to one csv file, which contains various metrics, observed parameters and classifier information.

### 2.2. Algorithms

Nodes and operations are the low and high-level algorithms in pySPACE (see Figure 1). They are organized in the missions package. New implementations have to be placed in the missions package and can then be used like the already implemented ones. Here, the type and granularity of input (as depicted in Figure 1) have to be considered, the algorithms need to inherit from the base class, and implement some basic processing function(s).

#### 2.2.1 Nodes

**Figure 2:**
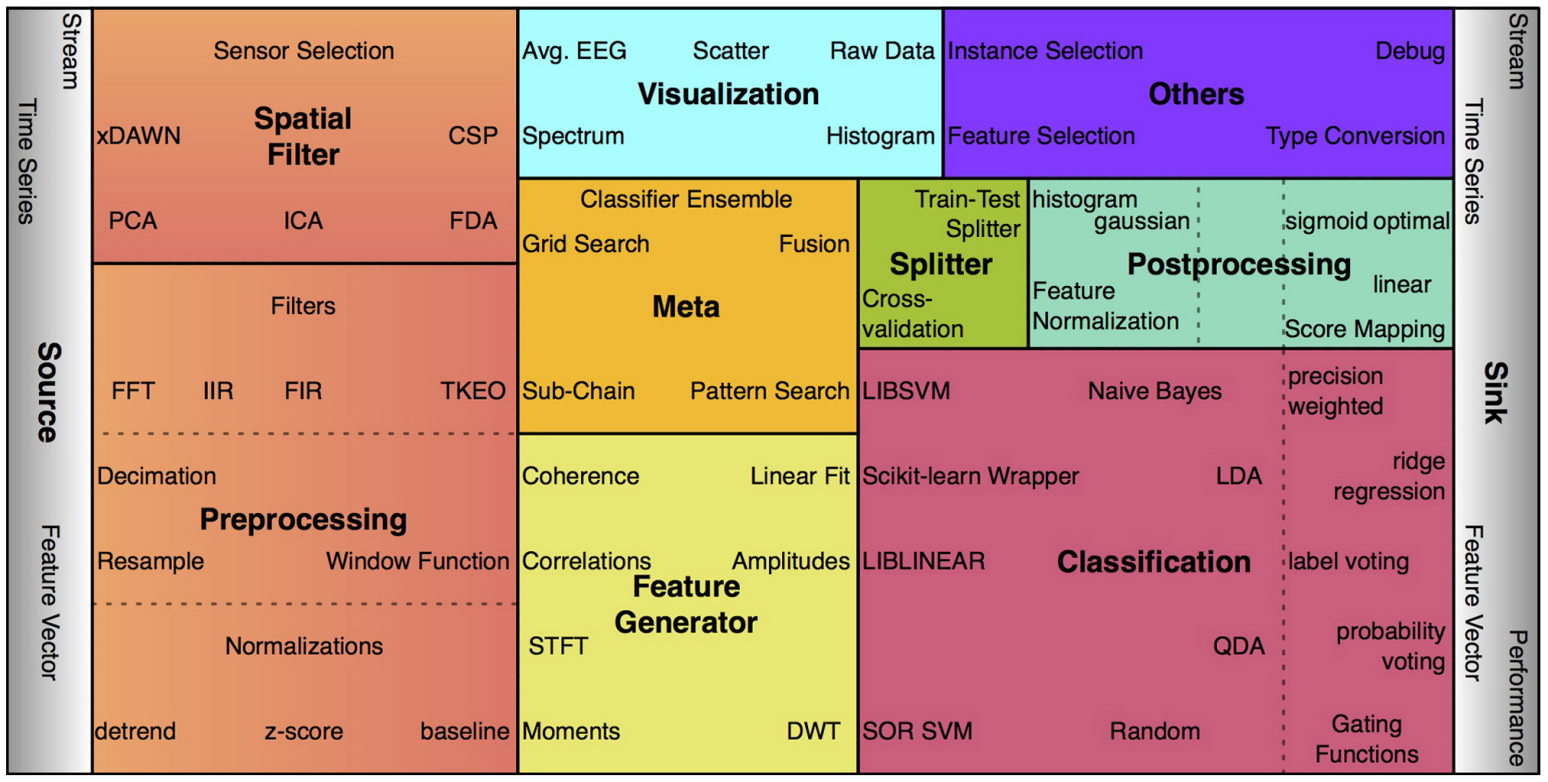
**Some examples of the more than 100 processing nodes in pySPACE, arranged according to processing categories (package names).** The size of the boxes indicates the respective number of currently available algorithms. For classification, postprocessing and preprocessing, subcategories are denoted.

The signal processing algorithms in pySPACE which operate on data samples (e.g., single feature vectors) are called nodes. Some nodes are trainable, i.e., they define their output based on the training data provided. The concept of nodes was inspired by Zito et al., (2008) as well as the concept of their concatenation, which is presented in section 2.3.1. Nodes are grouped depending on their functionality as depicted in Figure 2. Currently, there are more than 100 nodes available in pySPACE plus some wrappers for other libraries (MDP, LibSVM, Scikit-learn). A new node inherits from the base node and at least defines an execute function which maps the input (time series, feature vector, or prediction vector) to a new object of one of these types. Furthermore, it has a unique name ending with “Node” and its code is placed into the respective nodes folder. Templates are given to support the implementation of new nodes. For a complete processing of data from time series windows over feature vectors to the final predictions and their evaluation, several processing steps are needed as outlined in the following and in Figure 2.

*Preprocessing* comprises denoising time series data and reducing dimensionality in the temporal and frequency domain. By contrast, the *spatial filters* operate in the spatial domain to reduce noise. This can be done by combining the signals of different sensors to new virtual sensors or by applying sensor selection mechanisms. *Classification* algorithms typically operate on feature vector data, i.e, before classification the time series have to be transformed with at least one *feature generator* to a feature vector. A classifier is then transforming feature vectors to predictions. In *postprocessing*, feature vectors can be normalized and score mappings can be applied to prediction scores. For every data type a *visualization* is possible. Furthermore, there are *meta* nodes, which internally call other nodes or node chains. Thus, they can combine results of nodes or optimize node parameters. If training and testing data are not predefined, the data must be *split* to enable supervised learning. By default, data are processed as testing data.

*Source* nodes are necessary to request data samples from the datasets, *sink* nodes are required for gathering data together to get new datasets or to evaluate classification performance. They establish the connection from datasets to data samples which is required for processing datasets with concatenations of nodes.

#### 2.2.2. Operations

An operation automatically processes one data summary^2^ and creates a new one. It is also responsible for the mapping between summaries and datasets. Several operations exist for reorganizing data (e.g., shuffling or merging), interfacing to WEKA and MMLF, visualizing results or to access external code. The most important operation is, however, the node chain operation that enables automatic parallel processing of the modular node chain (see section 2.3.1). An operation has to implement two main functions. The first creates independent processes for specified parameter ranges and combinations, as well as different datasets. This functionality is the basis for the parallelization property of pySPACE (see section 2.3.3). The process itself defines the mapping of one or more datasets from the input summary to a dataset of the output summary and its call function is the important part. The second function of an operation is called “consolidate” and implements the clean up part after all its processes finished. This is especially useful to store some meta information and to check and compress the results. Operations and their concatenations are used for offline analysis (see section 3.3). In section 5.1 an example of an operation will be given and explained.

### 2.3 Infrastructure

So far we have discussed what to process (data) and which algorithms to use (nodes, operations). The infrastructure of pySPACE now defines how the processing is done. This core part is mainly defined in the environment package and usually not modified. It comprises the online execution (see section 3.4), the concatenation of nodes and operations (as depicted in Figure 1), and the parallel execution of processing tasks.

#### 2.3.1. Node chains

**Figure 3:**
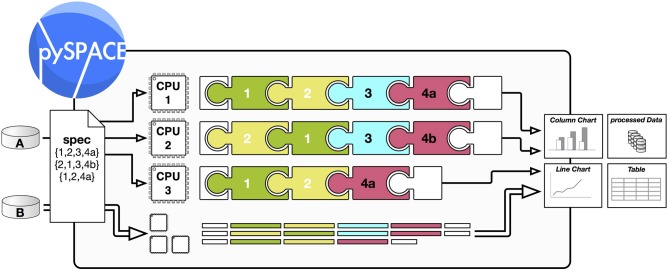
**Processing scheme of a node chain operation in pySPACE.** A and B are two different *datasets* (section 2.1), which shall be processed as specified in a simple *spec* file (section 3.2). The processing is then performed automatically. As a *result*, it can produce new data but also visualizations and performance charts. To speed up processing the different processing tasks can be *distributed* over several CPUs (section 2.3.3). The puzzle symbols illustrate different *modular nodes* (section 2.2.1), e.g., a cross-validation splitter (1), a feature generator (2), a visualization node (3), and two different classifiers (4a, 4b). They are concatenated to a *node chain* (section 2.3.1).

Nodes can be concatenated to a node chain to get a desired signal processing flow. The only restriction here is what a particular node needs as input format (raw stream data, time series, feature vector, or prediction vector). The input of a node chain is a dataset (possibly in an online fashion), which is accessed by a source node at the beginning of the node chain. For offline analysis, a sink node is at the end of the node chain to gather the result and return a dataset as output. In the online analysis, incoming data samples are processed immediately and the result is forwarded to the application. Between the nodes, the processed data samples are directly forwarded, and if needed cached for speed-up. Additional information can be transferred between nodes where this is necessary. To automatically execute a node chain on several datasets or to compare different node chains, a higher level processing is used: the node chain operation as depicted in Figure 3.

#### 2.3.2. Operation chains

Similar to concatenating nodes to node chains, operations can be concatenated to operation chains. Then, the first operation takes the general input summary and the others take the result summary of the preceding operation as input. At the end, the operation chain produces a series of consecutive summaries. Additionally to combining different operations, a benefit of the operation chain in combination with node chain operations is that a long node chain can be split into smaller parts and intermediate results can be saved and reused. In an operation chain, operations are performed sequentially so that parallelization is only possible within each operation.

#### 2.3.3. Parallelization

An offline analysis of data processing often requires a comparison of multiple different processing schemes on various datasets. This can and should be done in parallel to get a reduction of processing time by using all available CPUs. Otherwise, exhaustive evaluations might not be possible as they require too much time. *Operations* in pySPACE provide the possibility to create independent processes, which can be launched in a so-called “embarrassingly parallel” mode. This can be used for investigations where various different algorithms and parameters are compared (e.g., spatial filters, filter frequencies, feature generators). As another application example, data from different experimental sessions or different subjects might be processed in parallel. The degree of process distribution is determined in pySPACE by usage of the appropriate *back-end* for multicore and cluster systems. Figure 3 schematically shows how a data summary of two datasets is processed automatically with different node chains in parallel.

Additionally, some nodes of the meta package can distribute their internal evaluations by requesting own subprocesses from the back-end. This results in a two-level parallelization.

## 3. User and developer interfaces

pySPACE was designed as a complete software environment without requiring individual hand-written scripts for interaction. Users and developers have clearly defined access points to pySPACE that are briefly described in this section. Most of these are files in the YAML format (Ben-Kiki et al., 2008). Still, major parts of pySPACE can also be used as a library^3^, e.g., the included signal processing algorithms.

### 3.1. System and storage interface

The main configuration of pySPACE on the system is done with a small setup script that creates a folder, by default called *pySPACEcenter*, containing everything in one place the user needs to get started. This includes the global configuration file, links to main scripts to start pySPACE (see sections 3.3 and 3.4), a sub-folder for files containing the mission specification files (see section 3.2), and the data storage (input and output). Examples can be found in the respective folders. The global configuration file is also written in YAML and has default settings that can be changed or extended by the user.

### 3.2. Processing interface

**Figure 4:**
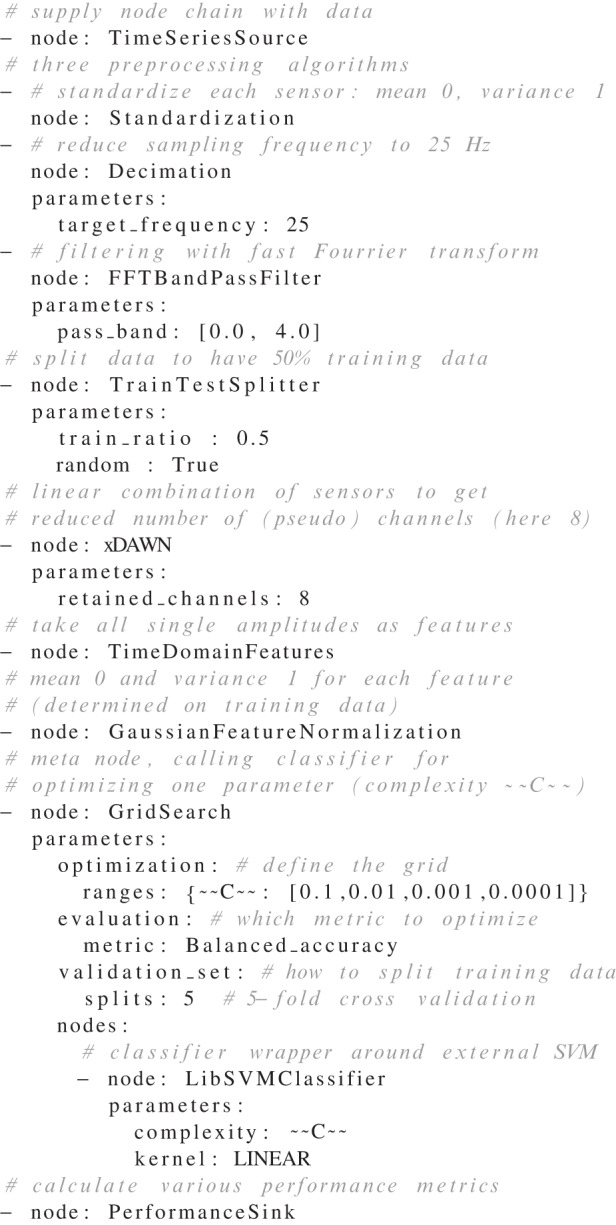
**Node chain example file.** Comments are denoted by a “#”. For further explanation see section 3.2.

**Figure 5:**
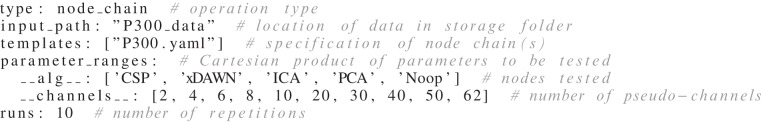
**Operation specification example file for spatial filter comparison.** For more details see discussion in section 5.1.

No matter if node chains, operations, or operation chains are defined (Figure 1), the specifications for processing in pySPACE are written in YAML. Examples are the node chain illustrated in Figure 4 or the operation illustrated in Figure 5. In addition to this file, the user has to make sure that the data are described with a short metadata file where information like data type and storage format are specified. If the data have been processed with pySPACE before, this metadata file is already present.

The types of (most) parameters in the YAML files are detected automatically and do not require specific syntax rules as can be inferred from the illustrated node chain (Figure 4), i.e., entries do not have to be tagged as being of type integer, floating point, or string. On the highest level, parameters can consist of lists (introduced with minus on separate lines like the node list) and dictionaries (denoted by “key: value” pairs on separate lines, or in the Python syntax, like {key1: value1, key2: value2}). During processing, these values are directly passed to the initialization of the respective object.

Figure 4 shows an example of a node chain specification that can be used to process EEG data. It illustrates the concatenation of different node categories (introduced in section 2.2.1)^4^. Data samples for this node chain could, e.g., consist of multiple EEG channels and multiple time points, so that after loading one would obtain windowed time series. Each data sample is then processed as specified: each channel is standardized, reduced in sampling rate and lowpass filtered. Then, the data are equally split into training and testing data to train the supervised learning algorithms, which are, in this example, the spatial filter xDAWN (Rivet et al., 2009), the feature normalization and the classifier later on [here, the LibSVM Support Vector Machine as implemented by Chang and Lin, (2011)]. Included in this node chain is a hyper-parameter optimization (grid search) of the complexity parameter of the classifier. This is done with five-fold cross-validation on the training data. Finally, performance metrics are calculated respectively for training and testing data. In a real application, the example in Figure 4 can be used to classify a P300 component in EEG data (Courchesne et al., 1977). More information on the paradigm and signal type is given elsewhere (Metzen et al., 2011b; Kirchner et al., 2013).

### 3.3. Offline analysis

Stored data can be analysed in pySPACE using the launch.py script. This script is used for operations and operation chains. The user only needs the respective specification file in YAML. The file name is a mandatory parameter of launch.py. For having non-serial execution but a distribution of processing, the parallelization mode parameter (e.g., “mcore” for multicore) is required. The operation specified in a file called my_operation.yaml can be executed from the command line, e.g., as


./launch.py -o my_operation.yaml --mcore.


**Figure 6:**
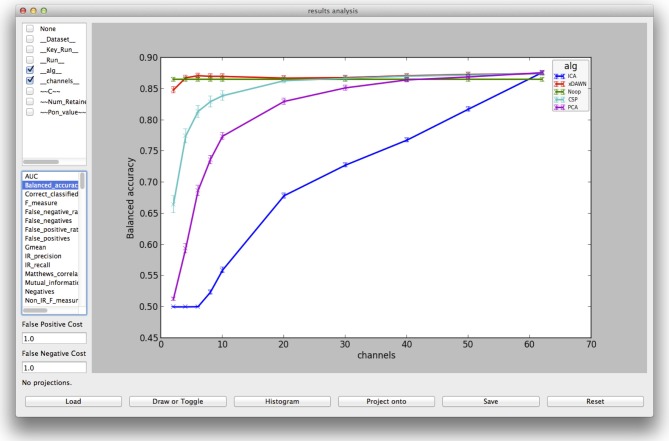
**Visualization from the evaluation GUI for the result of the spatial filter comparison, explained in section 5.1**.

Graphical user interfaces exist for construction of node chains and for exploration of the results. With the latter (example in Figure 6), different metrics can be displayed, parameters compared and the observation can be reduced to sub-parts of the complete results output, e.g., explore only results of one classifier type, though several different were processed. In section 5.1 an example of an offline analysis is given and explained.

### 3.4. Online analysis

For processing data from a recording device in an application, it is required to define a specific node chain, train it (if necessary) and then use it directly on incoming data. This is possible using the *pySPACE live* mode. It allows to define a certain application setup (such as involved components, communication parameters, acquisition hardware, number and type of node chains) by using additional parameter files that reference other pySPACE specification files (like in the offline analysis).

Several node chains can be used concurrently to enable simultaneous and parallel processing of different chains. For this, data are distributed to all node chains and the results are collected and stored or sent to the configured recipient (e.g., a remote computer). The data can be acquired from a custom IP-based network protocol or directly from a local file for testing purposes and simulation. Data from supported acquisition-hardware^5^ can be converted to the custom network protocol using a dedicated software tool, that comes bundled with pySPACE.

### 3.5. Developer interface

The documentation of pySPACE is designed for both, users and developers. It is automatically created with the documentation generator Sphinx^6^ combined with a customized generator of the documentation structure creating overviews of existing packages, modules and classes. For developers the source code of each described element is linked in the documentation.

Integration of new nodes, operations and dataset definitions is straightforward due to the modular nature of pySPACE. Once written and included in the software structure, they automatically appear in the documentation and can be used with the general YAML specification described above. If necessary, single nodes can be defined externally of pySPACE and they will still be included likewise, if they are specified via the global configuration file (section 3.1).

All operations and nodes come with a parameter description and a usage example. Additionally, test scripts and unit tests are available in the test component of pySPACE. The documentation is generated and unit tests are automatically executed on an everyday basis. For bug fixing, bug reports are possible via email to the pySPACE developer list or via issue reports on https://github.com/pyspace/pyspace.

## 4. Availability and requirements

pySPACE can be downloaded from https://github.com/pyspace and is distributed under GNU General Public License. The documentation can be found there, too. Currently supported operating systems are Linux and MacOSX. For parallelization, off-the-shelf multi-core PCs as well as cluster architectures using MPI or the IBM LoadLeveler system can be interfaced. The software requires Python2.6 or 2.7, NumPy, SciPy and YAML. Further optional dependencies exist, e.g., Matplotlib (Hunter, 2007) is required for plotting. Computational efficiency is achieved by using C/C++-Code libraries where necessary, e.g., SVM classification can be performed using the LIBSVM package.

## 5. Applications

pySPACE is applicable in various situations, from simple data processing over comprehensive algorithm comparisons to online execution. In this section an example for an offline analysis is given that comprises most of the key features of pySPACE. Thereby it is shown how the intended analysis can be easily realized without the need for programming skills. Furthermore, published work is named where pySPACE has been used, most often with such an offline analysis.

### 5.1. Example: algorithm comparison

In the following, an exemplary and yet realistic research question for processing neurophysiological data serves to explain how a node chain can be parameterized and thus different algorithms and parameters can be tested. To show that for such a comparison of algorithms and/or algorithm parameters pySPACE can be a perfect choice, the whole procedure from data preparation to final evaluation of the results is described.

#### 5.1.1. Data and research question

Let us suppose the following scenario: EEG data of 62 electrodes in an oddball paradigm were recorded [e.g., as described in Metzen et al., (2011b)] containing epochs of brain activity elicited by rare important stimuli (*targets*) and epochs of brain activity elicited by more frequent unimportant stimuli (*standards*). Our aim, besides the distinction of the two classes *Standard* and *Target*, is to investigate the effect of different spatial filters, i.e., ICA (Hyvärinen, 1999), PCA (Abdi and Williams, 2010), xDAWN (Rivet et al., 2009), and CSP (Blankertz et al., 2008), on the classification performance, or whether one should not use any spatial filter at all. Spatial filters aim to increase the signal-to-noise ratio by combining the data of the original electrodes to pseudo-channels. Thereby, not only performance can be increased, but also information is condensed into few channels, enabling reduction of dimensionality and thereby reducing the processing effort. Thus, a second research question here is to evaluate the influence of the number of pseudo-channels on the classification performance.

#### 5.1.2. Data preparation

In our example, each recording consists of five datasets. Since we want to randomly use half of the data for training and the remainder for estimating performance, the datasets of one recording have to be concatenated. This is an available *operation* in pySPACE after the data were transferred from stream (raw EEG format) to the pySPACE time series format. Therefore, after data preparation, all merged recordings that should be processed are present in the input path (see below), each in a separate sub-directory with its own meta file.

#### 5.1.3. Processing configuration

The algorithm comparison has to be specified in a file as depicted in Figure 5. The *type* keyword declares the intended *operation*, i.e., node chains will be executed. The data, which can be found in the directory P300_data (*input_path*) will be processed according to the specifications in the file P300.yaml. This file is identical to the one presented in Figure 4, except that it is parameterized to serve as a *template* for all node chains that should be executed. The parameterization is done by inserting unique words for all variables that need to be analyzed. This means, in the example that the specification of the xDAWN node is replaced by


- node : __alg__
  parameters :
    retained_channels : __channels__


introducing __alg__ and __channels__ as parameters. All values that should be tested for these two parameters are specified in the operation file (Figure 5) below the keyword *parameter_ranges*. pySPACE will create all possible node chains of this operation using the Cartesian product of the value sets (grid). The value of the parameter __alg__ is the corresponding node name, with Noop (meaning *No* *Op*eration) telling pySPACE that in this condition nothing should be done with the data. So Noop could serve as a baseline showing what happens when no spatial filter is used.

In the example, varying the number of retained channels will, in the case of Noop, lead to equal results for each value. Therefore, an additional constraint could ensure that Noop is only combined with one value of __channels__ and so reduce computational effort. Furthermore, instead of a grid of parameters, a list of parameter settings could be specified or Python commands could simplify the writing of spec files for users with basic Python knowledge. For example, the command range(2, 63, 2) could be used to define a list of even numbers from 2 to 62 instead of defining the number of retained pseudo-channels individually.

Finally, the *runs* keyword declares the number of repeated executions of each node chain. Repetitions can be used to compensate for random effects in the results due to components in the node chain that use randomness, like the *TrainTestSplitter*. To ensure reproducibility of the results, randomness in pySPACE is realized by using the random package of Python with a fixed seed that is set to the index of the repeated execution. In other words, the same value of *runs* returns the same results for a given data and operation. If one wants to obtain different results, this number has to be changed.

#### 5.1.4. Execution and evaluation

The execution works as described in section 3.3. The result is stored in a folder in the data storage, named by the time-stamp of execution. For replicability, it contains a zipped version of the software stack and the processing specification files. For each single processing result there is a subfolder named after the processed data, the specified parameters and their corresponding values. For evaluation, performance results are not stored separately in these single folders, but the respective metrics are summarized in a csv tabular. Furthermore, by default the result folders are also compressed and only one is kept as an example. The result visualization with the evaluation GUI of pySPACE can be seen in Figure 6. Here, the varied parameters (compare test parameters in Figure 5 with selection in upper left of Figure 6) as well as the data can be selected and individually compared with respect to the desired metric.

### 5.2. Published work

Since pySPACE became open source software in August 2013, there is not yet a public user community. Nevertheless, pySPACE has been developed and tested since 2008. The resulting publications show a small subset of possible applications of the software, documenting its applicability on EEG and EMG data (e.g., Kirchner and Tabie, 2013). In Kirchner et al. (2010), Wöhrle et al. (2013), Seeland et al. (2013), and Kirchner et al. (2013) pySPACE was used for evaluations on EEG data in the context of real applications. Some machine learning evaluations on EEG data were performed (Metzen and Kirchner, 2011; Metzen et al., 2011b; Kassahun et al., 2012). In Metzen et al. (2011a) and Ghaderi and Kirchner (2013) the framework is used for evaluation of spatial filters as also done in section 5.1. An example for a large-scale comparison of sensor selection algorithms can be found in Feess et al. (2013). Here, the parallelization in pySPACE for a high performance cluster was required, due to high computational load coming from the compared algorithms and the amount of data used for this evaluation. Recently, pySPACE was used for an evaluation of a new classifier on synthetic and benchmarking data Krell et al. (2013).

## 6. Related work

The Python machine learning stack is organized roughly starting from core libraries for numerical and scientific computation such as NumPy (Dubois, 1999) and SciPy (Jones et al., 2001), over libraries containing implementations of core machine learning algorithms such as Scikit-learn (Pedregosa et al., 2011) to higher level frameworks such as MDP (Zito et al., 2008), which allow to combine several methods and evaluate their performance empirically. Besides that, there are non-standardized ways of interfacing with machine learning tools that are not implemented in Python such as LibSVM (Chang and Lin, 2011) and WEKA (Hall et al., 2009). The distinction between libraries and frameworks is typically not strict; frameworks often contain some implementations of basic processing algorithms as libraries do and libraries typically include some basic framework-like tools for configuration and evaluation. pySPACE can be considered as a high-level framework which contains a large set of built-in machine learning algorithms as well as wrappers for external software such as Scikit-learn, MDP, WEKA, and LibSVM.

In contrast to libraries like Scikit-learn, the focus of pySPACE is much more on configuration, automation, and evaluation of large-scale empirical evaluations of signal processing and machine learning algorithms. Thus, we do not see pySPACE as an alternative to libraries but rather as a high-level framework which can easily wrap libraries (and does so already for several ones) and makes using and comparing the algorithms contained in these libraries easier.

In contrast to frameworks like MDP, pySPACE requires less programming skills since a multitude of different data processing and evaluation procedures can be completely specified using configuration files in YAML-syntax without requiring the user to write scripts, which would be a “show-stopper” for users without programming experience. Similarly, frameworks based on graphical user interfaces are not easily used in distributed computing contexts on remote machines without graphical interface. Thus, we consider pySPACE's YAML-based configuration files a good compromise between simplicity and flexibility.

Additionally, pySPACE allows to execute the specified experiments on different computational modalities in a fully automated manner using different back-ends: starting from a serial computation on a single machine, over symmetric multiprocessing on shared-memory multi-core machines, to distributed execution on high-performance clusters based on MPI or IBM's job scheduler LoadLeveler. Further back-ends like one integrating IPython parallel (Pérez and Granger, 2007) could easily be integrated in the future. Other tools for parallel execution are either restricted to the symmetric multiprocessing scenario like joblib (Varoquaux, 2013) or by themselves not directly usable in machine learning without some “glue” scripts such as IPython parallel.

A further advantage of pySPACE is that it easily allows transferring methods from the offline benchmarking mode to the processing in real application scenarios. The user can use the YAML-based data processing specifications in both modes.

There are several further open source signal processing toolboxes which could be interesting to be interfaced with pySPACE like OpenVibe (Renard et al., 2010), BCI2000 (Schalk et al., 2004), EEGLAB (Delorme and Makeig, 2004), Oger (Verstraeten et al., 2012), pyMVPA (Hanke et al., 2009), Shogun (Sonnenburg et al., 2010), and many more, including frameworks which would only use the automatic processing and parallelization capabilities of pySPACE. These interfaces might help to overcome some limitations of the software like the focus on feature vector and segmented time series data or the missing interactive data visualization. In the future, pySPACE would benefit from additional algorithms, input/storage formats, job distribution back-ends, and use cases. For example, the integration of video and picture processing could be a promising new use case. A broad scientific user community of pySPACE would provide a basis for easy exchange and discussion of signal processing and classification approaches, as well as an increased availability of new signal processing algorithms from various disciplines.

### Conflict of interest statement

The authors declare that the research was conducted in the absence of any commercial or financial relationships that could be construed as a potential conflict of interest.

## Footnotes

^1^published at previous Frontiers special issue “Python in Neuroscience.”

^2^Note that a summary can also consist of just a single dataset.

^3^This requires adding the pySPACE folder to the PYTHONPATH variable.

^4^For simplicity, most default parameters were not displayed.

^5^e.g., the BrainAmp USB Adapter by Brain Products GmbH (Gilching, Germany).

^6^http://sphinx-doc.org/
